# DualDistill: a dual-guided self-distillation approach for carotid plaque analysis

**DOI:** 10.3389/fmed.2025.1554578

**Published:** 2025-05-15

**Authors:** Xiaoman Zhang, Jiang Xie, Haibing Chen, Haiya Wang

**Affiliations:** ^1^School of Medicine, Shanghai University, Shanghai, China; ^2^School of Computer Engineering and Science, Shanghai University, Shanghai, China; ^3^Ultrasonic Center, Luodian Hospital, Shanghai, China; ^4^Department of Geriatrics, Shanghai Ninth People's Hospital, Shanghai Jiao Tong University School of Medicine, Shanghai, China

**Keywords:** ultrasound video classification, carotid plaque recognition, self-distillation, spatial-temporal attention, intra-frame relationship, deep learning

## Abstract

Accurate classification of carotid plaques is critical to assessing the risk of cardiovascular disease. However, this task remains challenging due to several factors: temporal discontinuity caused by probe motion, the small size of plaques combined with interference from surrounding tissue, and the limited availability of annotated data, which often leads to overfitting in deep learning models. To address these challenges, this study introduces a structured self-distillation framework, named DualDistill, designed to improve classification accuracy and generalization performance in analyzing ultrasound videos of carotid plaques. DualDistill incorporates two novel strategies to address the identified challenges. First, an intra-frame relationship-guided strategy is proposed to capture long-term temporal dependencies, effectively addressing temporal discontinuity. Second, a spatial-temporal attention-guided strategy is developed to reduce the impact of irrelevant features and noise by emphasizing relevant regions within both spatial and temporal dimensions. These strategies jointly act as supervisory signals within the self-distillation process, guiding the student layers to better align with the critical features identified by the teacher layers. Besides, the self-distillation process acts as an implicit regularization mechanism, which decreases overfitting in limited datasets. DualDistill is designed as a plug-and-play framework, enabling seamless integration with various existing models. Extensive experiments were conducted on 317 carotid plaque ultrasound videos collected from a collaborating hospital. The proposed framework demonstrated its versatility and effectiveness. It achieved consistent improvements in classification accuracy across 13 representative models. Specifically, the average accuracy improvement is 2.97%, with the maximum improvement reaching 4.74% on 3D ResNet50. These results highlight the robustness and generalizability of DualDistill. It shows strong potential for reliable cardiovascular risk assessment through automated carotid plaque classification.

## 1 Introduction

Cardiovascular and cerebrovascular diseases are important public health concerns that can lead to serious events such as stroke and heart attack. The accumulation of plaques atherosclerotic in the arteries is an important cause of these diseases (1). Therefore, early detection of carotid plaques is essential for timely intervention and prevention. Imaging modalities, such as magnetic resonance angiography (MRA), computed tomography angiography (CTA), and ultrasound (US), play a crucial role in guiding clinical decision-making. Among these, ultrasound (US) is widely utilized in medical imaging due to its non-invasive and real-time capabilities. To provide essential insights for risk assessment, carotid plaques are commonly classified as hyperechoic, mixed-echoic, or hypoechoic based on echogenicity (2). However, diagnostic accuracy among sonographers can be influenced by variations in experience and subjective interpretation. Therefore, incorporating artificial intelligence for diagnostic assistance is essential to enhance consistency and reliability in medical imaging.

With rapid advancements in deep learning, significant progress has been made in medical imaging tasks. As a result, research has increasingly focused on improving plaque recognition techniques through deep learning models. In 2017, a convolutional neural network (CNN) was proposed to automatically characterize carotid plaque composition in ultrasound images, demonstrating the feasibility of deep learning in this domain (3). To enhance feature extraction, a multi-path architecture was later introduced to detect plaques in OCT images using transfer learning and specialized data augmentation (4). Following this, a ResNet-based method for plaque classification was developed, utilizing a segmented region of interest (ROI) and transfer learning to improve performance (5). In 2022, a multi-feature fusion approach combined global ultrasound features, ROI echogenicity, and expert knowledge to improve high-risk plaque identification (6).

Deep learning in medical imaging, including carotid plaque analysis, is constrained by the need for large annotated datasets. Self-distillation has emerged as a promising solution, improving feature learning by using the model's own outputs. Self-distillation has been applied to 3D image segmentation tasks, enabling the model to learn both global semantic information and local spatial details simultaneously (7). In surgical instrument segmentation, self-distillation has also been used to extract knowledge from class probability maps. This helps reduce interference from unrelated information and improves segmentation accuracy (8). For thyroid nodule identification, a joint optimization strategy based on self-distillation was introduced. It combines high-level abstract features with multi-scale information, enhancing diagnostic precision (9). Additionally, an efficient self-distillation method was developed for early neoplasia detection. This approach uses high-level and low-level feature representations to address the challenges of limited training data (10). However, the aforementioned methods have not focused on carotid plaque identification using ultrasound imaging. For carotid plaque analysis, challenges arise due to indistinct boundaries and interference with blood flow, making feature extraction difficult. Additionally, ultrasound imaging itself has limitations, including lower resolution and higher susceptibility to noise compared to high-resolution modalities like MRI and CT. To the best of our knowledge, self-distillation has not yet been applied in the domain of ultrasound video analysis. However, compared to static ultrasound images, ultrasound videos contain richer spatial-temporal details (11, 12).

Deep learning has made great strides in video classification by extracting spatial-temporal features from video data. 2D Convolutional Neural Networks (2D CNNs) are commonly used for this task, where spatial features are extracted from individual frames and combined with temporal modeling techniques (13–15). However, separating the handling of temporal features limits the dynamic representation of video data. To overcome this, 3D Convolutional Neural Networks (3D CNNs) were introduced. These networks extract both spatial and temporal features directly from video sequences using 3D convolutions (16–18). More recently, transformer-based models have improved video classification by capturing complex spatial-temporal dependencies through self-attention mechanisms (19–23). Although these advances have proven to be effective for natural videos, the identification of carotid plaque in ultrasound videos presents unique challenges that differ significantly from typical video classification tasks.

**Figure 1:**
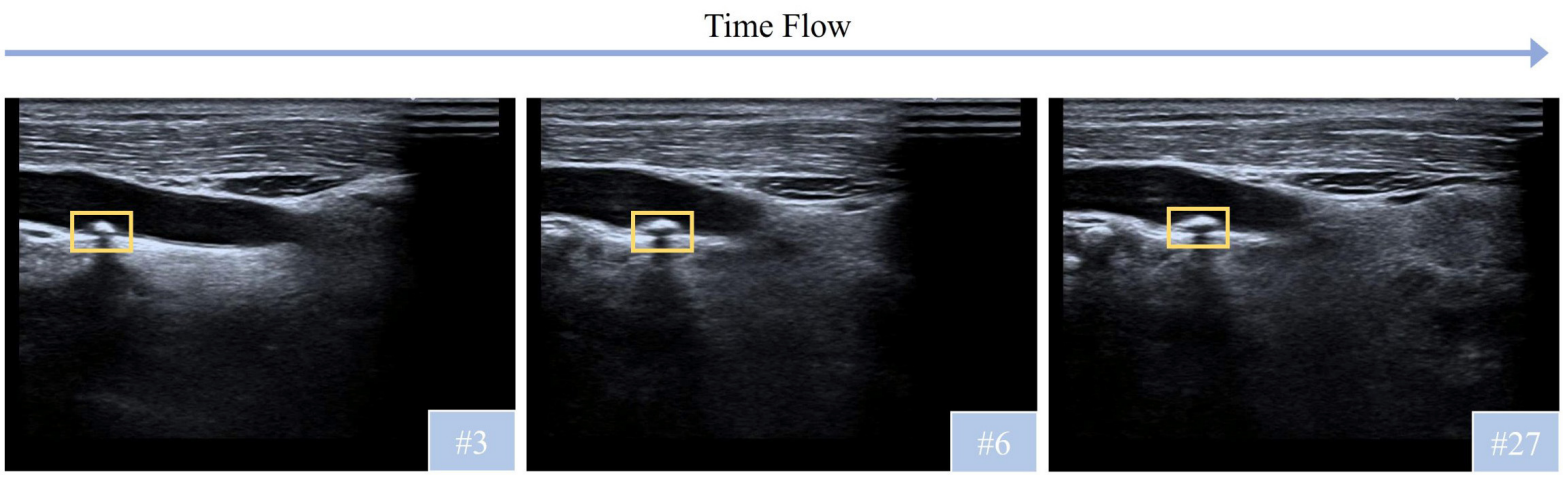
Temporal variation in plaque morphology observed through ultrasound video frames.

The characteristics of carotid plaques introduce additional complexity and pose additional challenges to plaque classification, as shown in Figure 1. (i) The 6th frame of the video demonstrates greater similarity to the 27th frame than to the nearer 3rd frame, due to the movement and adjustments of the probe during ultrasound examination. This phenomenon demonstrates challenges for continuity and consistency analysis in ultrasound videos, increasing the complexity of accurately detecting targets. (ii) Plaques are often small, morphologically irregular, and dynamically change over time. Combined with the inherent noise and low resolution of ultrasound, these factors require detection models capable of robust spatial-temporal feature learning and strong generalization under noisy conditions.

To tackle these challenges, researchers have started exploring various strategies to enhance the analysis of carotid plaque in ultrasound videos. Currently, the primary focus is on two main aspects: combining plaque tracking with classification or segmentation tasks and utilizing video-level annotations for plaque analysis. One line of research combines plaque tracking with classification or segmentation to improve overall accuracy. For example, Siamatsn improves both tracking and segmentation by performing these tasks simultaneously in the analysis of carotid plaques (24). Similarly, CPTV applied a tracking-based method to extract plaque features, while simultaneously leveraging ultrasound video characteristics to classify plaque echo types (12). Another direction of research addresses the burden of annotation by using only video-level annotations for plaque analysis. For example, RMFG_Net improved plaque area accuracy by fine-tuning target region features using a spatial-temporal attention block and integrating complementary features through a gated fusion model (25). Similarly, MA-Net aggregated three-dimensional temporal-channel-spatial features. It selectively focused on relevant frames, minimizing the influence of irrelevant information (26). Given the labor-intensive nature of annotating medical videos, this study focuses on video-level analysis. In this task, some research has already been conducted.

However, existing studies have two main limitations. First, temporal relationships between frames ofen are overlooked, which is crucial for resolving issues such as irregular plaque shapes. By learning the relationships between consecutive frames, models can better understand the progression and context of plaque formation, leading to more accurate detection despite shape inconsistencies. Second, there is an over-reliance on attention modules. While attention mechanisms can highlight important features, they may hinder generalization by making the model overly dependent on specific patterns in the training data. This reduces the model's robustness and limits its ability to perform well on unseen data due to dependency on specific data patterns.

This study proposes a dual-guided self-distillation approach called DualDistill to improve plaque classification performance using ultrasound videos. To the best of our knowledge, this is the first study to employ self-distillation in ultrasound video data. This approach incorporates two core strategies: relationship-guided self-distillation (RGSD) and attention-guided self-distillation (AGSD). From a local perspective, AGSD is employed to extract important information from ultrasound videos, effectively reducing interference and noise. Rather than directly adding attention modules, self-distillation distills attention information. This process improves model robustness, reduces computational complexity, and improves generalization. By allowing the model to iteratively refine its focus on key features, it better captures relevant patterns. From a global perspective, RGSD captures and incorporates temporal relationships across frames, allowing the model to learn long-term dependencies. These two components work in a complementary manner, ultimately contributing to the overall robustness and accuracy of plaque classification. The contributions of this study are mainly threefold:

- Relationship-guided self-distillation is developed to capture temporal dependencies, helping the model identify key patterns across frames and reduce the impact of inconsistencies.
- Attention-guided self-distillation is designed to focus on the most crucial regions across thereby minimizing the impact of redundant or irrelevant information.
- DualDistill is proposed to integrate these two strategies effectively mitigates temporal inconsistencies while enhancing feature focus, leading to improved classification performance. And DualDistill exhibits strong generalization capabilities, delivering consistent performance improvements across diverse model architectures.

## 2 Methodology

**Figure 2:**
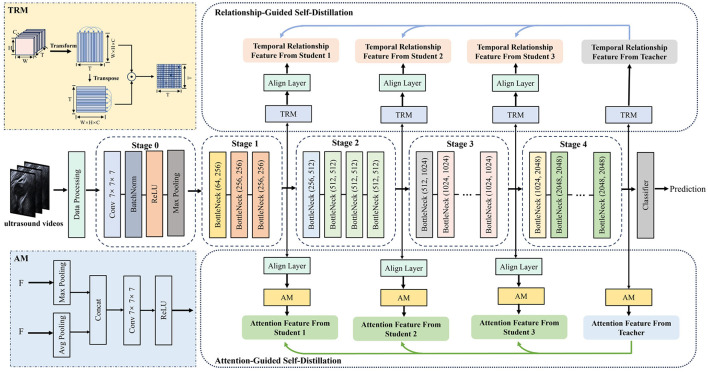
Overview of the proposed DualDistill framework, detailing the Relationship-Guided Self-Distillation and Attention-Guided Self-Distillation processes for ultrasound video analysis. AM represents the Attention Module, and TRM refers to the Temporal Relationship Module.

In this section, DualDistill is proposed to achieve accurate plaque echo classification in carotid ultrasound videos as shown in Figure 2. The approach comprises two strategies: RGSD and AGSD. In RGSD, Temporal Relationship Module (TRM) is employed to capture the relational information from different frames. During training, the deepest layer's output acts as the teacher, guiding shallower layers to learn effective feature representations. In AGSD, spatial and temporal attentions are generated simultaneously by the Attention Module (AM). This module utilizes average-pooling and max-pooling operations to generate two attention maps, which are then combined through concatenation and processed by a convolutional layer. Subsequently, the self-distillation mechanism transfers the knowledge of spatial-temporal attention from the deepest layer to the shallower layers. In addition, a fundamental self-distillation method is employed, which extracts knowledge from soft labels and intermediate features to enhance robustness and generalizability.

### 2.1 Self-distillation

Given a training set of $X=\left\{x_{1},x_{2},...,x_{n}\right\}$ and the corresponding set of labels $Y=\left\{y_{1},y_{2},...,y_{n}\right\}$, each sample $x_{i}\in {\mathbb{R}}^{T\times C\times W\times H}$ is a ultrasound video of carotid plaque. Then, the carotid plaque aims to find a classifier *c* that maps the ultrasound to its corresponding label. Specifically, assuming *c* = *g*°*f* where *g* is the classification head and *f* is the neural network backbone for feature encoding, then we can formulate *f* = *f*_*K*_°*f*_*K*−1_°…*f*_1_, where *K* denotes the number of convolutional stages in *f* and *f*_*i*_ denotes the convolutional stage *i*_*th*_. In each convolution stage, self-distillation attaches an auxiliary classifier *g*_*i*_. Thus, there are *K* classifiers, which can be written as


(1) $$\begin{array}{l} \;c_{1}(x)=g_{1}{}^{\circ}f_{1}(x),\\ \;c_{2}(x)=g_{2}{}^{\circ}f_{2}{}^{\circ}f_{1}(x),\\ \;\ldots \\ c_{K}(x)=g_{K}{}^{\circ}f_{K}{}^{\circ}f_{K-1}{}^{\circ}\ldots {}^{\circ}f_{1}(x) \end{array}$$

By denoting the well-known cross-entropy loss and Kullback-Leibler divergence as $L_{\text{CE}}$ and $L_{\text{KL}}$, respectively, the training loss of the original self-distillation (27) can be formulated as


(2) $$\begin{array}{l} {ℒ}_{\text{SD}}=\sum_{K}^{i=1}{ℒ}_{\text{CE}}\left(c_{i}\left(X),Y\right)\right)\\ \;+\alpha \cdot \sum_{K-1}^{i=1}{ℒ}_{\text{KL}}(c_{i}(X),c_{K}(X)) \end{array}$$

where the first item is the original training loss of each classifier and the second item is the self-distillation loss. α is a hyper-parameter to balance them. In this mechanism, the output of the deepest layer serves as the teacher, while the outputs of the shallower layers act as students, guiding the latter to learn more efficient feature representations.

### 2.2 Relationship-guided self-distillation

While self-distillation lays the foundation for enhancing model robustness, the relationship-guided self-distillation introduces a more specific approach by capturing temporal dependencies across frames. In the task of plaque classification, the temporal relationships between different frames in a video contains valuable semantic information. And the variability in probe positioning and adjustments during ultrasound examinations poses significant challenges in maintaining continuity and consistency in ultrasound video analysis. Therefore, RGSD is designed to address the shortcomings in capturing long-term temporal dependencies. It also helps mitigate the challenges posed by irregular plaque morphology. In each selected convolutional stage, TRM is employed to generate efficient representations for temporal relationships. For simplicity, we denote the feature at the *i*_*th*_ stage as $F_{i}=f_{i}•f_{i-1}•\cdots •f_{1}\in {\mathbb{R}}^{T\times C\times W\times H}$ where *T, C, W, H* denotes the number of frames, the number of channels, the width and height of each video, respectively. The proposed relationship-guided self-distillation firstly flattens the features across the spatial dimensions, resulting in *G*∈ℝ^*B*×*T*×(*W*·*H*·*C*)^. The flattening process retains contextual information across frames, thereby ensuring that the relationships are preserved. Then, the temporal relationships can be formulated as


(3) $$\begin{array}{l} R=\frac{G\cdot G^{t}}{\left||G||||G^{t}|\right|} \end{array}$$

where the script *t* indicates the operation of matrix transpose, *R*∈ℝ^*T*×*T*^ is the matrix of temporal relationships, and *G*^*t*^ is the transpose of the matrix *G*. Additionally, ||*G*|| denotes the Frobenius norm of matrix *G*, which is defined as:


$$||G||=\sqrt{\underset{i,j}{\sum }G_{ij}^{2}}$$

By distinguishing the temporal relationships of features at different convolutional stage with the script *i*, then the loss function of the proposed temporal relationships can be formulated as


(4) $$\begin{array}{l} L_{\text{RGSD}}=\overset{K-1}{\underset{i}{\sum }}||R_{i}-R_{K}|{|}_{2} \end{array}$$

where $R_{i}\in {\mathbb{R}}^{T\times T}$ denotes the temporal relationship features from the *i*-th intermediate stage, *R*_*K*_ corresponds to the final-stage relationship feature, and ||·||_2_ computes the element-wise Euclidean distance between *R*_*i*_ and *R*_*K*_.

### 2.3 Attention-guided self-distillation

While RGSD captures temporal relationships between frames, attention-guided self-distillation (AGSD) refines the model's focus on critical features. In carotid ultrasound videos, accurate feature extraction is challenged by noise. Therefore, dynamically modulating attention across both temporal and spatial dimensions not only improves the extraction of key features but also reduces the model's sensitivity to noise. Thus, the AGSD mechanism is designed to emphasize the relevant information in both spatial and temporal dimensions. The simultaneous extraction of temporal and spatial attention enables the model to capture both dynamic changes and static features. This enhances the model's ability to understand the full context of the data. Additionally, this approach helps mitigate the challenges posed by low-quality images, as it allows the model to focus on the more relevant information despite noise or interference.

As depicted in Figure 2, AM was designed to generate spatial-temporal attention maps. Given an input F, two feature maps are generated by two pooling operations, average pooling and max pooling: $F_{avg}^{ST}\in {\mathbb{R}}^{1\times T\times H\times W}$ and $F_{max}^{ST}\in {\mathbb{R}}^{1\times T\times H\times W}$. Max-pooling emphasizes the most salient local regions within the feature map, while average pooling provides global contextual information. To effectively preserve both local and global contextual information, these two feature maps are concatenated directly. Then the concatenated features are subjected to additional processing via a convolutional layer, leading to the generation of the final output. In short, the spatial-temporal attention is computed as:


(5) $$\begin{array}{l} M=f^{7\times 7}([AvgPool(F);MaxPool(F)])\\ \;=f([F_{avg}^{ST};F_{max}^{ST}]) \end{array}$$

where *f*^7 × 7^ represents a convolution operation with the filter size of 7 × 7. Then the loss function of the proposed spatial temporal attention can be formulated as


(6) $$\begin{array}{l} L_{\text{AGSD}}=\overset{K-1}{\underset{i}{\sum }}||M_{i}-M_{K}|{|}_{2} \end{array}$$

where $M_{i}\in M^{T\times H\times W}$ denotes the spatitial-temporal attention map from the *i*-th intermediate convolutional stage, *M*_*K*_ represents the final-stage attention map, and ||·||_2_ calculates the Euclidean distance between them.

### 2.4 Overall loss function

In summary, the proposed dual-guided self-distillation (DualDistill) framework enhances the traditional self-distillation (SD) approach by integrating both temporal dependencies and attention-based mechanisms. The general loss function effectively combines these components, thus optimizing model performance in ultrasound video analysis. To sum up, the overall loss function can be formulated as


(7) $$\begin{array}{l} L_{\text{overall}}=L_{\text{SD}}+\beta \cdot L_{\text{RGSD}}+\gamma \cdot L_{\text{AGSD}} \end{array}$$

where β and γ are hyper-parameters to balance the three loss functions. The experiments for sensitivity and ablation study are shown in Figure 4, Table 1. Moreover, the proposed approach primarily functions during training and do not engage in the inference stage, thus preventing the addition of extra parameters.

**Table 1:** Ablation study of the three modules in DualDistill: SD, RGSD, and AGSD.

**SD**	**RGSD**	**AGSD**	**Accuracy**	**Sensitivity**	**Specificity**	**Precision**	**F1-Score**	**Time (hours)**
			84.75 ± 0.72	84.97 ± 0.68	84.64 ± 0.77	84.84 ± 0.63	85.94 ± 0.58	1.03
✓			86.65 ± 0.71	87.32 ± 0.64	85.97 ± 0.74	86.81 ± 0.59	87.54 ± 0.54	1.13
	✓		86.34 ± 0.63	86.59 ± 0.58	86.22 ± 0.68	86.71 ± 0.54	87.73 ± 0.49	1.08
		✓	87.00 ± 0.59	87.42 ± 0.54	86.79 ± 0.64	87.23 ± 0.49	89.27 ± 0.44	1.10
✓	✓		87.61 ± 0.54	88.22 ± 0.49	87.00 ± 0.58	87.79 ± 0.44	88.91 ± 0.39	1.16
✓		✓	87.35 ± 0.69	87.71 ± 0.44	87.17 ± 0.74	88.30 ± 0.49	88.52 ± 0.44	1.17
	✓	✓	87.52 ± 0.64	87.84 ± 0.39	87.36 ± 0.48	87.79 ± 0.34	88.30 ± 0.28	1.15
✓	✓	✓	**89.50 ± 0.38**	**89.73 ± 0.33**	**89.39 ± 0.43**	**89.64 ± 0.27**	**90.57 ± 0.23**	1.22

Time refers only to the training time. Bold values denote the highest performance scores for each metric (e.g., accuracy, F1-score).

## 3 Experiments and results

### 3.1 Dataset

**Figure 3:**
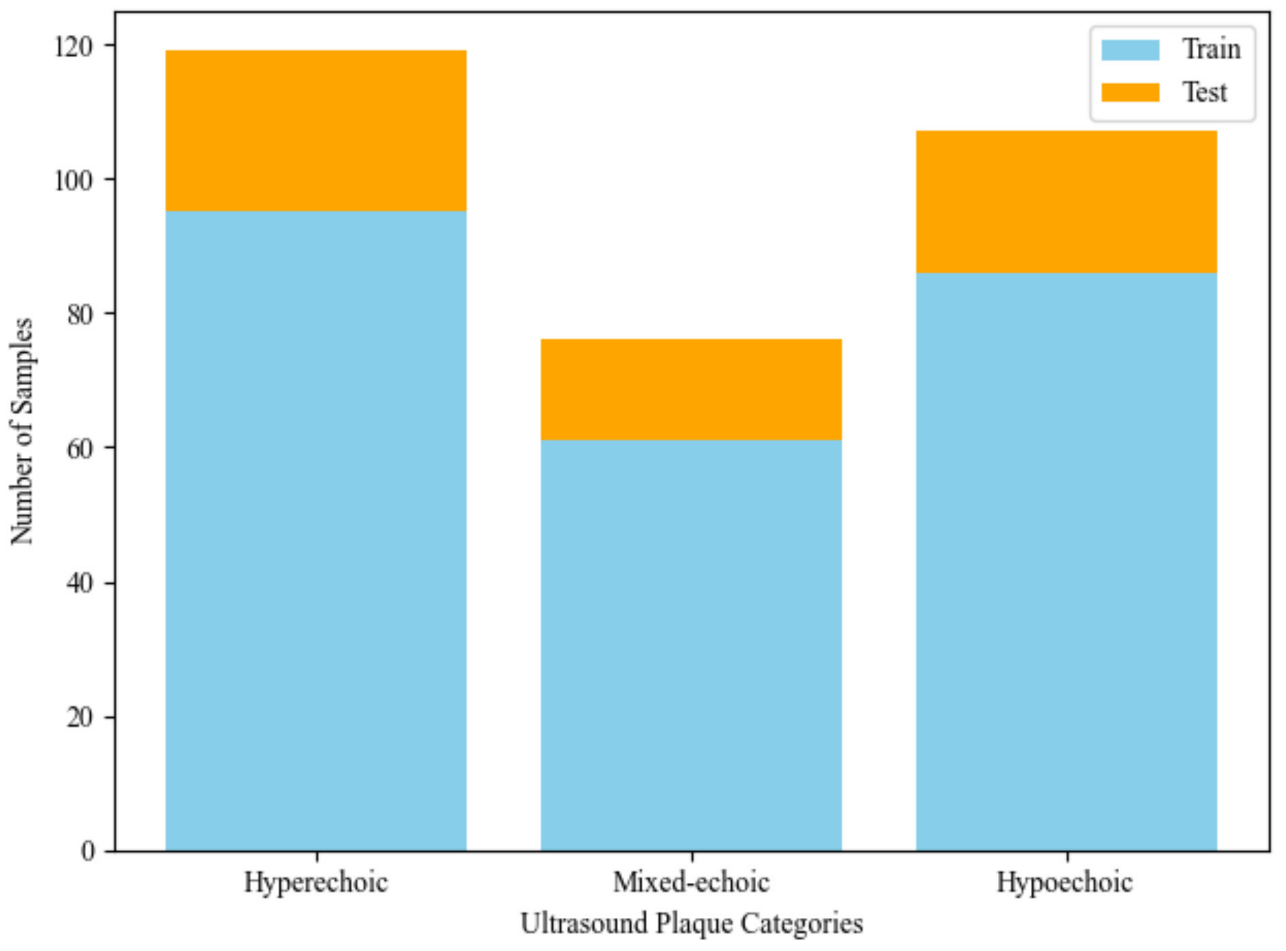
Distribution of plaque types across training and test sets in the ultrasound video dataset.

A total of 317 carotid plaque ultrasound videos are obtained from a collaborating hospital. This study was carried out in close collaboration with medical experts from our collaborating hospitals, with senior clinicians guiding the data annotation process to ensure the study aligns with real-world diagnostic practices. Each video comprises 120 frames, with a resolution of 740 × 540 pixels. The dataset includes patients aged 57-85 years (mean 68.2 ± 10.5 years), with a balanced gender ratio (1.1:1 male-to-female). As shown in Figure 3, the dataset consists of 317 ultrasound videos categorized into three classes: 113 videos of mixed-echoic plaques, 124 hyperechoic plaques, and 80 hypoechoic plaques. To ensure the robustness of the evaluation, a 5-fold cross-validation technique is employed. The dataset was divided into training and testing sets in an 8:2 ratio. The labels were annotated by a senior sonographer with over 10 years of clinical experience, incorporating evaluations of echogenicity (hyperechoic, hypoechoic, mixed-echoic). All ultrasound data is acquired in B-mode using a MyLab Twice system equipped with a 7-13 MHz transducer.

### 3.2 Implementation details

In the experimental implementation, two data augmentation techniques were applied: random resized cropping to increase dataset variability and horizontal flipping with a 50% probability to improve the model's adaptability to different orientations. During the training phase, the input video data is pre-processed by resizing each frame to 224 × 224 pixels and normalizing the pixel values using the mean and variance of the Kinetics400 dataset. The learning rate is initially set to 0.001 and subsequently adjusted using a step decay schedule, with a tenfold reduction applied at specified epochs. Moreover, a linear warm-up strategy is employed during the first 10 epochs to gradually increase the learning rate to 0.001. Optimization is performed using Stochastic Gradient Descent (SGD), configured with a momentum of 0.9 and a weight decay of 0.0001. In addition, gradient clipping is applied to enhance training stability. To assess the performance of each model, accuracy, precision, sensitivity, specificity, and F1-score were computed. The average results across the five folds are reported to provide a comprehensive evaluation of model generalization capabilities.

### 3.3 Model evaluation techniques

Evaluation metrics are crucial to assess model performance in video classification tasks. Accuracy measures the proportion of correctly classified instances, providing an overall effectiveness measure. Sensitivity evaluates the model's ability to identify positive instances, vital in minimizing false negatives, such as in medical diagnostics. Precision calculates the proportion of true positives among predicted positives, which is critical in reducing false positives in applications such as fraud detection. Specificity, on the other hand, measures the proportion of true negatives among the predicted negatives, which is important in minimizing false positives and is crucial in scenarios like disease screening. To balance precision and sensitivity, the F1-score, their harmonic mean, is especially useful for imbalanced datasets. Furthermore, the AUC-ROC curve, which plots the true positive rate against the false positive rate, provides an aggregate measure of a model's ability to distinguish between classes across various thresholds. Together, these metrics provide a comprehensive understanding of the strengths and limitations of the model. These four metrics are defined as follows:


(8) $$\begin{array}{l} \text{Accuracy}=\frac{\text{TP}+\text{TN}}{\text{TP}+\text{TN}+\text{FP}+\text{FN}} \end{array}$$

(9) $$\begin{array}{l} \text{Sensitivity}=\frac{\text{TP}}{\text{TP}+\text{FN}} \end{array}$$

(10) $$\begin{array}{l} \text{Specificity}=\frac{\text{TN}}{\text{TN}+\text{FP}} \end{array}$$

(11) $$\begin{array}{l} \text{Precision}=\frac{\text{TP}}{\text{TP}+\text{FP}} \end{array}$$

(12) $$\begin{array}{l} \text{F1-score}=2\cdot \frac{\text{Precision}\cdot \text{Sensitivity}}{\text{Precision}+\text{Sensitivity}} \end{array}$$

(13) $$\begin{array}{l} \text{True Positive Rate}=\frac{\text{TP}}{\text{TP}+\text{FN}} \end{array}$$

(14) $$\begin{array}{l} \text{False Positive Rate}=\text{Sensitivity} \end{array}$$

where True Positives (TP) are correctly classified positives, True Negatives (TN) are correctly classified negatives, False Positives (FP) are negatives misclassified as positives, and False Negatives (FN) are positives misclassified as negatives.

### 3.4 Ablation study

To evaluate the contribution of the RGSD and AGSD modules to the model's performance, a comprehensive ablation study was conducted. RGSD and AGSD are the two core strategies in the proposed DualDistill, which aims to enhance plaque classification performance using ultrasound videos. AGSD, from a local perspective, is employed to extract important features from the ultrasound videos, effectively reducing noise and interference. RGSD, from a global perspective, captures and incorporates temporal relationships across frames, allowing the model to learn long-term dependencies.

In the ablation study, the independent application of RGSD and AGSD led to accuracy improvements of 1.59% and 2.25%, respectively, highlighting their individual contributions to model performance. AGSD demonstrated more consistent improvements, reflected by its lower standard deviation (0.59%) compared to RGSD (0.63%). When combined with self-distillation, RGSD and AGSD improved accuracy by 1.01% and 0.75%, respectively, compared to self-distillation alone. Additionally, when applied together without self-distillation, RGSD and AGSD achieved a 2.87% accuracy improvement, surpassing the individual performance of RGSD and AGSD by 1.18% and 0.52%, respectively. This suggests that their complementary application results in more effective performance enhancement.Finally, integrating all three components–RGSD, AGSD, and SD (self-distillation) resulted in significant improvements across all metrics: accuracy increased by 4.74%, sensitivity by 4.76%, precision by 4.80%, and F1-score by 4.63%. These results show that combining RGSD and AGSD with self-distillation significantly enhances the model's overall performance. Each distillation loss independently improves the model by focusing on different aspects: RGSD enhances sensitivity by targeting the positive class, while AGSD improves specificity by reducing false positives. Together, they complement each other, leading to better performance.

### 3.5 Trade-off analysis

Using all three modules–RGSD, AGSD, and SD–presents the most effective approach when considering both performance improvements and training time. The combination of these modules results in the highest accuracy increase of 4.74%, as each module contributes uniquely to the model's overall performance. In terms of training time, while the use of all three modules adds 0.19 hours to computational cost (from 1.03 to 1.22 hours), this is a modest trade-off considering the notable performance gain. Moreover, the increased training time only impacts the training phase and does not affect inference speed or model size. Therefore, the slight increase in training time is justified by the substantial improvement in accuracy. The balance between performance enhancement and training time makes the use of all three modules the most suitable and efficient choice for achieving optimal results in practical applications.

### 3.6 Sensitivity Study

#### 3.6.1 Sensitivity study on Hyper-parameters

**Figure 4:**
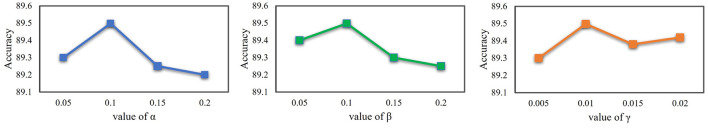
Sensitivity analysis of hyperparameters for 3D ResNet-50.

As introduced in Equation 2 and Equation 7, three hyperparameters α, β and γ are introduced in this paper to balance the magnitudes of different loss functions. The 3D ResNet-50 backbone was used for this analysis, and results are shown in Figure 4. It was observed that even with suboptimal hyperparameter values, the accuracy dropped by only 0.3% while remaining 4.4% higher than the baseline model, indicating that our method is robust to the choice of hyperparameters.

#### 3.6.2 Sensitivity study on which stages

This section focuses on the effect of selecting different student feature positions in the self-distillation process. In this setup, feature positions within the 3D ResNet-50 architecture can be conveniently integrated into 4 convolutional stages by residual blocks. The output of the deepest residual block acts as the teacher, while various combinations of earlier stages are selected as student layers. The choice of these student layers has an impact on the model's overall performance, as shown in Table 2.

**Table 2:** Sensitivity analysis of student feature positions across different stages.

**Stages**	**Accuracy**	**Sensitivity**	**Specificity**	**Precision**	**F1-score**
1, 4	86.38 ± 0.73	86.65 ± 0.67	86.25 ± 0.76	86.62 ± 0.64	87.01 ± 0.59
2, 4	86.38 ± 0.69	87.00 ± 0.71	85.94 ± 0.81	86.77 ± 0.69	86.93 ± 0.66
3, 4	87.65 ± 0.63	87.49 ± 0.61	87.73 ± 0.56	87.42 ± 0.57	87.97 ± 0.51
1, 2, 4	87.88 ± 0.56	87.78 ± 0.49	87.93 ± 0.61	87.81 ± 0.53	88.66 ± 0.46
1, 3, 4	87.79 ± 0.61	87.47 ± 0.54	87.95 ± 0.64	87.73 ± 0.59	88.44 ± 0.54
2, 3, 4	88.27 ± 0.52	88.27 ± 0.46	88.27 ± 0.51	88.49 ± 0.47	89.21 ± 0.41
**1, 2, 3, 4**	**89.50 ± 0.38**	**89.73 ± 0.33**	**89.64 ± 0.43**	**89.64 ± 0.27**	**90.57 ± 0.23**

Bold values denote the highest performance scores for each metric (e.g., accuracy, F1-score).

This analysis shows that the proposed method achieves optimal results when stages 1, 2, 3, and 4 are selected as student layers. Increasing the number of stages involved in the self-distillation process consistently enhances performance. Furthermore, among configurations with the same number of stages, deeper stages (closer to the teacher) outperform those farther away. This is because features from stages closer to the teacher align more closely with the teacher's high-level representations, allowing the student layers to capture the teacher's complex abstractions more effectively. In addition to improved performance, the model using all stages exhibits the lowest variance across all metrics. This indicates more consistent results and fewer fluctuations in performance. On the other hand, configurations with fewer stages show higher variability, suggesting that using fewer stages leads to less stable learning and generalization. Thus, the variance analysis reinforces the advantage of incorporating more stages in the self-distillation process for more reliable and robust model performance.

### 3.7 AUC-ROC curve analysis

**Figure 5:**
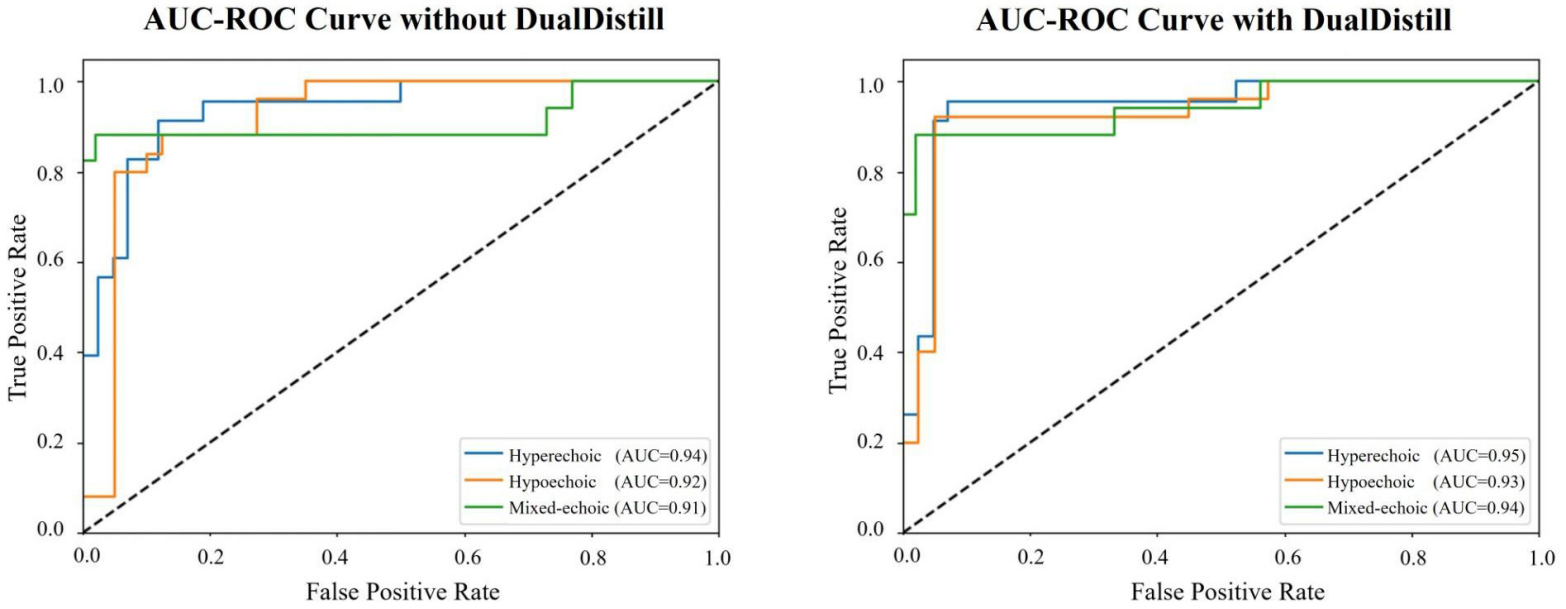
AUC-ROC curves of 3D ResNet50 with vs. without DualDistill for carotid plaque video classification.

As shown in Figure 5, the AUC values for all three categories–hyperechoic, hypoechoic, and mixed-echoic–are higher when DualDistill is applied. Specifically, the AUC for hyperechoic improves from 0.94 to 0.95, hypoechoic from 0.92 to 0.94, and mixed-echoic from 0.91 to 0.94. Mixed-echoic plaques are difficult to classify due to their combination of hyperechoic and hypoechoic features. This overlap makes them harder to distinguish from stable (hyperechoic) and vulnerable (hypoechoic) plaques. The significant performance boost for mixed-echoic plaques demonstrates the robustness of our approach in handling these complex cases and improving the accuracy of plaque stability classification. Additionally,the AUC-ROC curves with DualDistill are closer to the top-left corner, indicating better sensitivity and specificity. These results demonstrate that DualDistill enhances the model's ability to classify carotid plaque conditions more effectively and consistently across all categories.

### 3.8 Visualization

As previously addressed, mixed-echoic plaques pose significant challenges in identification. DualDistill demonstrates a more substantial improvement in recognizing mixed-echoic plaques compared to other types of plaque. To further assess the effectiveness of the two modules, visualization analysis was performed on two mixed-echoic plaque samples, evaluating how DualDistill effectively focuses on key information and learns temporal relationships between frames.

**Figure 6:**
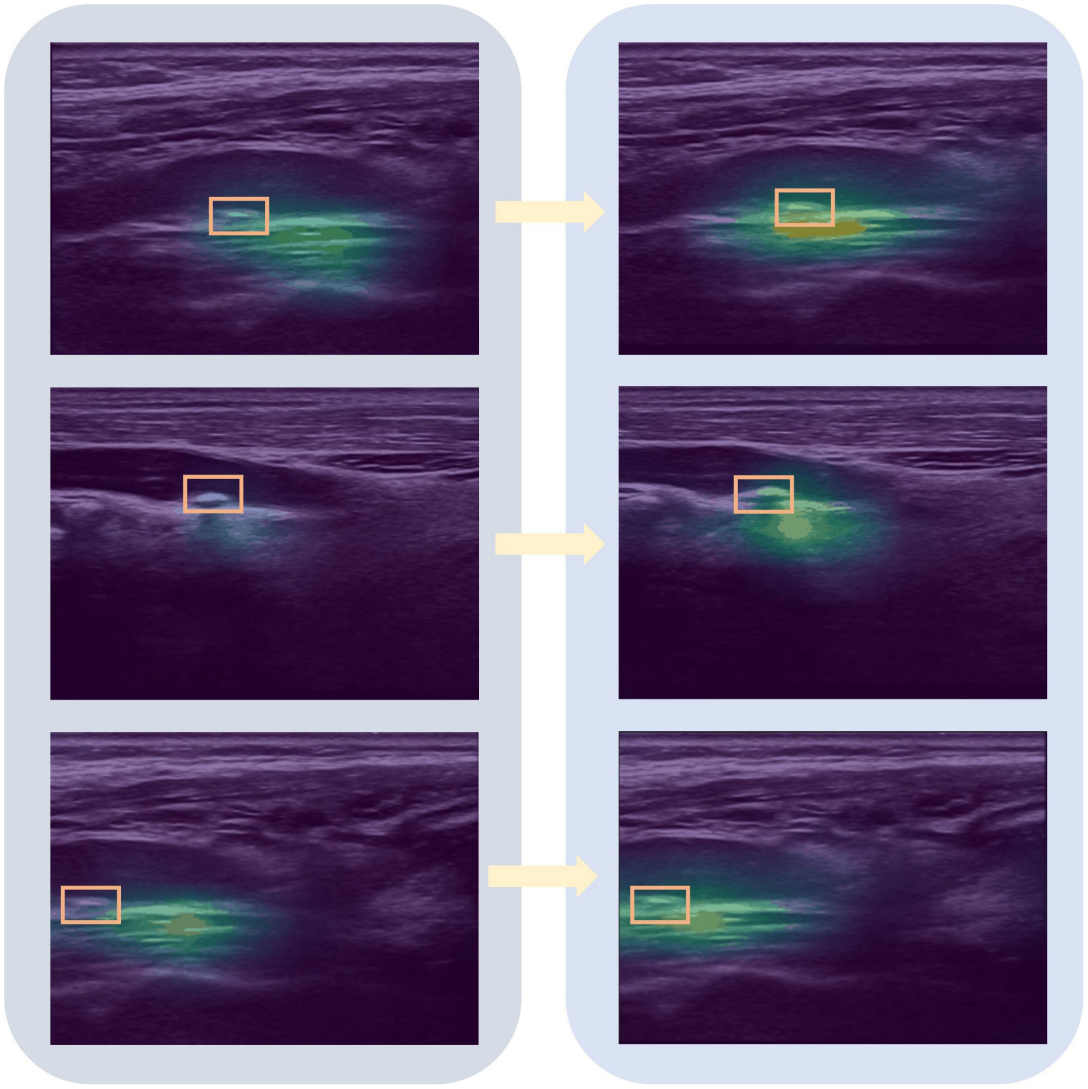
Grad-CAM attention map visualization on a mixed-echoic sample: comparison between results without DualDistill **(left)** and with DualDistill **(right)**.

The Grad-CAM visualization (28) shown in Figure 6 illustrates that DualDistill effectively alleviates localization ambiguity in this mixed-echoic plaque sample. This figure presents a direct comparison between models with and without DualDistill. It shows that the model with this method can better localize the critical plaque regions. This indicates that this approach improves the model's ability to capture relevant spatial information during classification. In contrast, the model without the proposed method exhibits more diffuse and inconsistent focus, likely contributing to its reduced classification accuracy.

**Figure 7:**
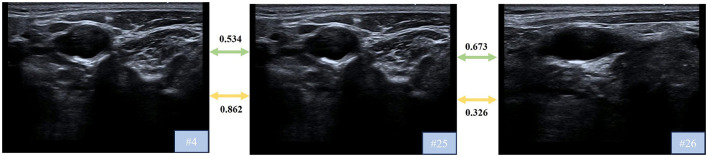
Frame-to-frame relationship coefficients on a mixed-echoic sample: without DualDistill (green lines) vs. with DualDistill (yellow lines).

The frame-to-frame relationship coefficients are normalized to a range between -1 and 1, where positive values denote higher similarity between frames, while negative values indicate a divergence. Figure 7 presents an analysis of frame-to-frame relationship coefficients for three selected frames (the 4th frame, the 25th frame, and the 26th frame) from an ultrasound video sequence. It can be observed that, although there is a substantial temporal gap between the 4th and 25th frames, their similarity remains high, whereas the 25th and 26th frames, despite being consecutive, exhibit lower similarity. Following the application of the proposed approach, the similarity coefficient between the 4th and 25th frames increased significantly, from 0.534 to 0.862. Conversely, the similarity coefficient between the 25th and 26th frames decreased, from 0.673 to 0.326. These results highlight the effectiveness of the proposed method in capturing and refining the inherent relationships between frames, aligning the coefficients more closely with the observed temporal and visual dynamics.

### 3.9 Effectiveness analysis of DualDistill across models

The proposed structured self-distillation approach is implemented on a range of classic models to conduct comprehensive experimental evaluations. The experimental results presented in Table 3 illustrate that the proposed approach leads to significant performance improvements across all tested models. For the 2D convolutional models, TSM's accuracy increased from 82.82% to 86.24%, with similarly substantial gains observed for TIN, TSN and MA-Net. In the case of 3D convolutional models, C3D's accuracy improved from 83.25% to 87.05%, while SlowFast and C3D both showed marked enhancements. Furthermore, DualDistill also boosted the performance of Transformer-based models, such as TimesFormer, VideoMAE, VideoMAEv2, VideoSwin and CAST, demonstrating its effectiveness and robustness across different model architectures. The experimental results demonstrate that DualDistill significantly improves the model's performance, leading to an accuracy increase ranging from a minimum of 1.5% to a maximum of 4.05%. This consistent improvement across various settings highlights the effectiveness and robustness of the proposed approach in enhancing plaque classification accuracy. More importantly, these models trained with DualDistill all have lower variance across all metrics ranging from a minimum of 0.25% to a maximum of 0.79%. DualDistill enhances both the accuracy and stability of models, making it a valuable approach for improving carotid plaque ultrasound video classification. The method is architecture-agnostic, allowing for easy integration into existing clinical decision support systems. Since DualDistill is applied only during the training phase and does not alter the model structure or inference complexity, it has no impact on inference speed or FPS. The consistent performance improvement, without increasing inference time, ensures good clinical applicability.

**Table 3:** Performance comparison of different models on the carotid plaque ultrasound video dataset with and without DualDistill.

**Model**	**Accuracy**	**Sensitivity**	**Specificity**	**Precision**	**F1-score**
C3D (17)	83.25 ± 0.83	83.07 ± 0.79	83.34 ± 0.87	82.87 ± 0.77	82.48 ± 0.73
Our C3D	**87.05 ± 0.62**	**83.96 ± 0.67**	**88.60 ± 0.75**	**86.24 ± 0.59**	**87.76 ± 0.55**
TSN (15)	80.75 ± 0.81	82.43 ± 0.78	79.91 ± 0.85	81.84 ± 0.74	82.94 ± 0.66
Our TSN	**82.25 ± 0.74**	**83.35 ± 0.65**	**81.70 ± 0.79**	**83.64 ± 0.63**	**84.57 ± 0.58**
TIN (13)	83.21 ± 0.68	84.20 ± 0.63	82.72 ± 0.71	83.64 ± 0.57	84.67 ± 0.53
Our TIN	**85.35 ± 0.57**	**86.33 ± 0.55**	**84.86 ± 0.63**	**85.87 ± 0.55**	**86.58 ± 0.49**
TSM (14)	82.82 ± 0.71	83.13 ± 0.66	82.67 ± 0.76	82.82 ± 0.61	84.40 ± 0.56
Our TSM	**86.24 ± 0.55**	**85.29 ± 0.59**	**86.72 ± 0.65**	**86.32 ± 0.54**	**87.72 ± 0.50**
SlowFast (18)	84.10 ± 0.63	84.40 ± 0.58	83.95 ± 0.67	84.63 ± 0.53	86.41 ± 0.47
Our SlowFast	**87.35 ± 0.48**	**87.33 ± 0.45**	**87.36 ± 0.54**	**87.87 ± 0.44**	**89.58 ± 0.39**
X3D (16)	84.45 ± 0.59	84.62 ± 0.55	84.37 ± 0.65	85.45 ± 0.50	85.60 ± 0.45
Our X3D	**88.50 ± 0.43**	**87.73 ± 0.40**	**88.89 ± 0.49**	**87.64 ± 0.38**	**88.57 ± 0.35**
TimesFormer(19)	82.13 ± 0.69	82.34 ± 0.64	82.03 ± 0.74	82.86 ± 0.60	83.45 ± 0.54
Our TimesFromer	**85.75 ± 0.56**	**85.69 ± 0.50**	**85.78 ± 0.59**	**86.62 ± 0.45**	**87.70 ± 0.40**
VideoMAE (20)	81.26 ± 0.72	81.34 ± 0.68	81.22 ± 0.78	82.87 ± 0.65	82.46 ± 0.59
Our VideoMAE	**85.23 ± 0.60**	**85.93 ± 0.55**	**84.88 ± 0.66**	**85.32 ± 0.56**	**86.65 ± 0.50**
VideoSwin (22)	81.96 ± 0.70	81.14 ± 0.66	82.37 ± 0.75	83.35 ± 0.60	82.46 ± 0.55
Our VideoSwin	**84.24 ± 0.65**	**85.49 ± 0.50**	**83.62 ± 0.60**	**86.30 ± 0.45**	**85.23 ± 0.39**
VideoMAEv2 (21)	83.32 ± 0.65	82.74 ± 0.60	83.81 ± 0.69	83.36 ± 0.56	83.76 ± 0.50
Our VideoMAEv2	**85.76 ± 0.49**	**86.42 ± 0.45**	**85.43 ± 0.55**	**86.13 ± 0.40**	**87.32 ± 0.36**
CAST (23)	82.45 ± 0.59	83.24 ± 0.55	82.06 ± 0.65	83.34 ± 0.50	82.47 ± 0.45
Our CAST	**84.24 ± 0.55**	**86.36 ± 0.49**	**83.18 ± 0.60**	**86.76 ± 0.45**	**85.63 ± 0.39**
MA-Net (26)	87.36 ± 0.45	87.58 ± 0.40	87.25 ± 0.49	88.64 ± 0.35	87.84 ± 0.29
Our MA-Net	**88.96 ± 0.39**	**89.32 ± 0.35**	**88.78 ± 0.45**	**90.04 ± 0.29**	**89.42 ± 0.25**

Bold values denote the highest performance scores for each metric (e.g., accuracy, F1-score).

## 4 Conclusions

In this article, a structured self-distillation approach named DualDistill is proposed to improve the classification of carotid plaque in ultrasound videos, utilizing two key strategies: Relationship-guided Self-distillation (RGSD) and Attention-guided Self-distillation (AGSD). In RGSD, Temporal Relationship Module (TRM) is introduced to capture temporal relationships between frames, allowing the model to learn long-term dependencies by transferring high-level relational knowledge from deeper to shallower layers. In AGSD, Attention Module (AM) is developed to generate spatial-temporal attention maps, with attention knowledge distilled in the same way. This enhances the model's ability to focus on crucial regions and minimize irrelevant information. To the best of our knowledge, this is the first study to apply self-distillation to carotid plaque classification in ultrasound video data. To validate the effectiveness of the AGSD and RGSD, ablation studies were conducted. The results show that the best performance is achieved when both approach are applied on base of the self-distillation method. we performed sensitivity analysis experiments on both hyperparameters and student feature positions across different stages. These analyses provided valuable insights into the optimal configuration of the proposed approach. To further assess our approach, we evaluated it using AUC-ROC curve analysis and visualization techniques, including Grad-CAM. Specifically, these analyses demonstrate that DualDistill achieves the most significant improvement on the challenging task of identifying mixed-echoic plaques. The visualization results further indicate that DualDistill enables better extraction of key local information and facilitates learning of temporal relationships between frames. Moreover, DualDistill consistently improved classification performance across 13 representative models, underscoring its potential to enhance both accuracy and stability. DualDistill is architecture-agnostic, enabling easy integration into clinical decision support systems. Furthermore, it introduces only a modest increase in training time, with no impact on testing time. These factors make DualDistill particularly well-suited for clinical environments, where both high performance and operational efficiency are essential.

In future work, we plan to further refine the knowledge transfer process within self-distillation to better leverage the features within the model and optimize the training process. We also aim to establish collaborations with more hospitals to collect a broader set of multi-center data, which will help develop models with improved generalizability. Additionally, we intend to track patient information and gather more multimodal data, such as pathological information, to enhance the model's ability to understand complex clinical contexts and improve diagnostic accuracy. Moreover, we are developing related software to assist doctors in making more accurate diagnoses and plan to further validate and optimize our work in clinical settings.

## Data Availability

The data analyzed in this study is subject to the following licenses/restrictions: The dataset used in this study are subject to certain access restrictions due to patient privacy concerns. Requests to access these datasets should be directed to Xiaoman Zhang, zhangxiaoman1998@shu.edu.cn.
